# The Effect of Hyperbaric Oxygen Therapy on Kidneys in a Rat Model

**DOI:** 10.1155/2014/105069

**Published:** 2014-08-10

**Authors:** Matitiahu Berkovitch, Roni Tsadik, Eran Kozer, Ibrahim Abu-Kishk

**Affiliations:** Pediatric Division, Pediatric Intensive Care Unit, Assaf HaRofeh Medical Center, Affiliated to the Sackler School of Medicine, Tel-Aviv University, 70300 Zerifin, Israel

## Abstract

*Background*. Hyperbaric oxygen therapy (HBOT) is used for treating various medical conditions. As far as known yet, HBOT is safe with few major side effects that are easy to avoid using a proper protocol. Renal tubular damage was observed in rats exposed to HBOT in a preliminary study conducted in our institution. *Aim*. We aim to assess whether HBOT causes renal damage and, if so, whether this is dose dependent. *Methods*. Thirty-one rats were randomly assigned to three groups. The first group received 10-days HBOT, 100% oxygen at a pressure of 2 atmospheres absolute (2 ATA) for 60 minutes/day, the second received the same treatment for 5 days and the third served as the control. Rat weight, survival, renal function tests, and renal histopathology were analyzed. *Results*. There were no significant changes in renal function tests in the plasma (cystatin C, urea, creatinine, and electrolytes) between the groups. No significant differences were observed in weight gain or renal histopathological evaluation between all groups. *Conclusion*. HBOT in this protocol does not cause renal impairment in a rat model, which reinforces the assumption that HBOT is safe in healthy rats, regarding renal function. Further research should be focused on the effect/safety of HBOT on nonhealthy kidneys.

## 1. Introduction

Hyperbaric oxygen therapy (HBOT) is gaining popularity for the treatment of many medical indications. HBOT—breathing 100% oxygen at a pressure above the atmospheric pressure at sea level (>1 atmosphere absolute (ATA), 760 mmHg)—can result in increased arterial and tissue oxygen tension [1]. The main physiological effect of HBOT is an increase in the amount of oxygen dissolved in the plasma to a level sufficient to support tissues with minimal need for extracting the oxygen carried by hemoglobin. Such doses of oxygen have a number of beneficial biochemical, cellular, and physiological effects [1, 2].

A few studies have shown that HBOT is capable of inducing cellular protection against ischemia. These cellular protective mechanisms are still poorly understood, but studies have shown that they include the expression of stress-inducible proteins, including the antiapoptotic protein Bcl-2 and the free radical scavenger Mn-super oxide dismutase [3].

HBOT may have beneficial effects on the kidneys. One study has demonstrated that HBOT attenuated the increase in plasma creatinine, the deterioration in glomerular filtration rate (GFR), and the histopathologic damage in rats subjected to renal ischemia/reperfusion injury [4].

Many of the side effects of HBOT are known, such as barotraumas. Seizures can also be one of the side effects of HBOT. However, HBOT is considered safe in most of the cases when 100% oxygen is administered at a pressure < 3 ATA [1].

HBOT has been used successfully to treat some renal medical conditions. However, there is evidence of renal impairment when HBOT is used in combination with nephrotoxic drugs. For example, in one study, cisplatin caused more renal damage when HBOT was administered [5].

A study performed in our center examined the effect of HBOT treatment on renal functions in a rat model. The renal damage was induced by Amphotericin B intoxication [6]. However, renal histopathological findings compatible with acute tubular necrosis (ATN) were observed in three out of five subjects in the control group of rats treated with HBOT alone during the pilot phase. These histopathological findings had no correlation with renal function tests and they included degeneration with vacuolization of the tubular epithelium with tubular lumen obstruction suggesting ATN. The role of HBOT as a cause of these histopathological findings is unclear.

The HBOT protocol in that study consisted of administration of 100% oxygen at 2 ATA for 70 minutes, including 5 minutes of compression and 5 minutes of decompression time. The HBOT session used in the current study was similar to the usual sessions used in human subjects. For example, HBOT is administered at 2 ATA for periods of 60 minutes in patients with acute carbon monoxide poisoning [7]. Another HBOT protocol used in patients with traumatic brain injury includes 40 treatment sessions (5 days/week), 60 minutes each, with 100% oxygen at 1.5 ATA [8].

In this study, we aimed to examine whether HBOT may cause renal damage in a rat model and, if so, whether this damage is dose dependent.

## 2. Materials and Methods

This study was approved by the Assaf HaRofeh Medical Center Animal Care Committee. Male Sprague-Dawley rats weighing 220–270 g were studied. The rats were handled according to the guidelines of the Local Ethics Committee for Animal Experimentation.

Thirty-one young male Sprague-Dawley rats were randomly assigned to three groups. The primary outcome was ATN. In a previous study [6], three out of five rats treated with HBOT demonstrated ATN. Assigning 10 rats to each one of the three study groups yields a power of 80% to detect ATN in four out of 10 with an Alpha of 0.05. The first group (*n* = 10) received 10 days of HBOT in a 2 ATA hyperbaric chamber for 60 minutes. The second group (*n* = 10) received the same treatment for 5 alternating days, and the third (*n* = 11) served as the control group.

### 2.1. Hyperbaric Oxygen Administration

Hyperbaric oxygen was administered using a purpose-built animal hyperbaric chamber manufactured locally by our team of medical engineers. Treatment consisted of administration of 100% oxygen at 2 ATA for 70 minutes, including 5 minutes of compression and 5 minutes of decompression time.

The weight of the rats was measured every 5 days, and survival was evaluated. At day 10, all the rats were sacrificed and blood samples were drawn. The kidneys were removed for histopathological examination. The main outcome measure was kidney pathology changes. Secondary outcome measures were plasma cystatin C, urea, creatinine, sodium, potassium, and overall survival.

### 2.2. Biochemical Analysis

Measurements of plasma creatinine, urea, and magnesium were performed using the Roche/Hitachi modular P800 autoanalyzer (Roche Diagnostics, Mannheim, Germany). Evaluation of plasma potassium was performed using the Roche/Hitachi modular ISE 900 autoanalyzer (Roche Diagnostics, Mannheim, Germany). Acute kidney injury was defined as a doubling of creatinine levels in the plasma, and acute kidney failure was defined as a tripling of creatinine levels in the plasma [9, 10]. Cystatin C was evaluated by the Roche* Cobas Mira* instrument (Hoffman-La Roche Ltd., Basel, Switzerland) using a commercial kit, Dako Cytomation cystatin C Immunoparticles Set (DAKO, Hamburg, Germany) based on particle-enhanced immunoturbidimetry.

### 2.3. Pathological Evaluation

Following blood withdrawal, all the rats were sacrificed by cervical dislocation; their kidneys were removed, preserved in formaldehyde, and subsequently embedded with paraffin. Pathological samples were made perpendicular to the renal capsule, containing cortex and medulla. Samples were then stained by hematoxylin and eosin. The histopathologic examination was performed blindly by one expert pathologist. Estimation of the percentage of tubular necrosis, tubular lumen obstruction, and vacuolization (cell degeneration) was performed in three high power fields of affected area.

The main examined variable was renal histopathological changes, and secondary outcomes included changes in values of creatinine, urea, plasma electrolytes, and plasma cystatin C levels. Weight gain and rat survival were also recorded.

### 2.4. Statistical Analysis

Statistical analysis was performed using SPSS (ver. 19). The quantitative measurements are presented using their means, SDs, median, min, and max. Data was checked for normal distribution by the Shapiro-Wilk test. The ANOVA test was performed to examine whether there was a significant difference between the groups. Values were considered statistically significant when *P* < 0.05.

## 3. Results

During the experimental study, all the rats survived and gained weight by 20% on average with no significant differences between the groups (*P* = 0.112).  Table 1 summarizes the renal function tests, showing no significant differences between the groups. We could not identify renal histopathological changes such as cell swelling or focal tubular epithelial necrosis and apoptosis with desquamation of cells into lumen or dilated proximal tubules or signs of epithelial regeneration (flattened epithelium, dilated tubular lumina, large nuclei with prominent nucleoli, and mitotic activity) in any of the groups.

## 4. Discussion

The HBOT protocols proposed in the current study revealed no significant differences between the groups in terms of rat survival, weight gain, renal function indices, and renal histopathological changes. Since none of the rats treated with HBOT demonstrated renal damage, the possibility of a type two error is very low.

Recently, studies have been performed in order to investigate the efficacy of HBOT for other medical conditions such as cerebral or myocardial ischemia, fibromyalgia, traumatic brain injury, and more [2, 11–14]. However, there are known complications associated with HBOT such as barotraumas and neurological oxygen toxicity manifested by generalized seizures, even in patients without any recognizable risk factors [15]. Renal toxicity was also described in a rat model. In a previous study, rats were exposed to HBOT at either 4.8 ATA for 60 min or 6.8 ATA until the onset of convulsions. Only rats that suffered from HBOT-induced seizures were found to have alterations in renal function [16]. However, the exact mechanism of renal impairment is not clear. It is possible that the renal injury was induced by rhabdomyolysis due to status epilepticus rather than HBOT [17]. Another study that evaluated the effect of HBOT on renal functions in septic rats had surprising findings in the mean of the GFR values, even though the HBOT group (HBOT alone) and the septic group (septic without HBOT) separately showed decreased GFR. The addition of HBOT in the septic group ameliorated the GFR values [18]. The authors speculated that HBOT may have different effects on healthy and damaged kidneys. Another study found that HBOT reduced neutrophil infiltration after ischemia reperfusion injury and also limited lipid peroxidation by increasing antioxidant enzymes. Although the exact mechanisms of HBOT renal benefits were not clear, the authors speculated that the beneficial effects of HBOT were mediated via inhibiting neutrophil infiltration [19].

In this study, we examined indirect markers of GFR: creatinine, urea, and blood electrolytes. These chemical markers are easy to obtain, cheap, readily available, and used in common medical practice. When comparing different techniques of measuring GFR in acute kidney failure, measurement of plasma creatinine is not the optimal method. When GFR declines, the excretion of creatinine rises so in fact its plasma level is diminished which in turn causes an overestimation of the GFR [20]. Several investigators studied the feasibility of plasma cystatin C as a marker of glomerular filtration rate and found it to be more accurate than creatinine measurements [21]. In order to ameliorate the accuracy of renal function, plasma cystatin C levels were measured in our study and no significant differences were observed among the various groups.

When oxygen is breathed at high partial pressures, a hyperoxic condition will rapidly spread, with the most vascularized tissues being most vulnerable. Under environmental stress, levels of free reactive oxygen radicals may increase dramatically, which can damage cell structures and produce oxidative stress [22]. High concentrations of oxygen may also increase the production of other free radicals, such as nitric oxide, peroxynitrite, and trioxidane, which harm DNA and other biomolecules [22–24]. Although the body has many antioxidant systems, such as glutathione, which protect from oxidative stress, these systems are eventually overwhelmed at very high concentrations of free oxygen, and the rate of cell damage exceeds the capacity of the systems to prevent or repair it. Thus, cell damage and cell death result [25–27]. However, it appears that the administration of 100% oxygen at 2 ATA for 70 minutes used in the current study was insufficient to produce cell damage, even if administered consecutively for 10 days. The intervals between the HBOT administrations may have allowed time for the antioxidative system to recover.

Limitations of this study include the short period of follow-up and the use of few markers of renal function tests. However, the significance of the study lies in its offering of further assurance of the safety of HBOT treatment. We assume that the current findings may be useful for human subjects also, since the HBOT protocol used in the current study is similar to those used in human subjects.

In conclusion, HBOT in this protocol does not cause renal impairment in a rat model. The results of this research study reinforce the assumption that HBOT treatment is safe in healthy rats, with respect to renal function. Further research should be focused on the effects/safety of HBOT on nonhealthy kidneys.

## Figures and Tables

**Table 1:** Comparison of renal function tests between the groups (plasma measured).

Measured value	Group 1	Group 2	Control group	*P* value
Urea (mg/dL)	40.7 ± 6	42.4 ± 5	39.5 ± 7.8	0.596
Creatinine (mg/dL)	0.39 ± 0.04	0.36 ± 0.04	0.36 ± 0.03	0.142
Sodium (mmol/L)	141.2 ± 3.2	142.1 ± 2.5	141.9 ± 2.1	0.735
Potassium (mmol/L)	5.2 ± 1.3	5.5 ± 1.3	5 ± 1	0.597
Cystatin C (mg/L)	2.2 ± 0.44	1.92 ± 0.33	2.11 ± 0.37	0.23
